# The Prevention and Management of Contrast-induced Acute Kidney Injury: A Mini-review of the Literature

**DOI:** 10.7759/cureus.3284

**Published:** 2018-09-11

**Authors:** Asad Ali, Chandur Bhan, Muhammad Bilal Malik, Malik Qistas Ahmad, Shahzad Ahmed Sami

**Affiliations:** 1 Medicine, CMH Lahore Medical College and Institute of Dentistry, Lahore, PAK; 2 Internal Medicine, Chandka Medical College Hospital, Larkana, PAK; 3 Internal Medicine, Shifa College of Medicine, Islamabad, PAK; 4 Hematology/Oncology, University of Arizona Cancer Center, Tucson, USA; 5 Internal Medicine, CMH Lahore Medical College and Institute of Dentistry, Lahore, PAK

**Keywords:** nephropathy, acute tubular necrosis, pharmacologic treatment, nephrotoxicity, contrast induced nephropathy, hemodialysis, radiological, volume expansion, acute kidney injury, nephroprotective

## Abstract

Contrast-induced acute kidney injury, also called contrast-induced nephropathy, is one of the main causes of acute renal failure/acute kidney injury (AKI) in hospitalized patients within 48 to 72 hours of contrast media administration during various radiologic procedures. Several factors can be responsible for contrast-induced acute tubular necrosis (ATN); however, patient and procedure-related factors play the lead role in determining the development of contrast-induced nephropathy. There is no definitive treatment and hydration remains the mainstay preventive strategy. This article will review the incidence, criteria for definitive diagnosis, and an effective approach on how to prevent contrast-induced nephropathy in a clinical setup.

## Introduction and background

Contrast-induced acute kidney injury, also called contrast-induced nephropathy, is an abrupt deterioration in renal function following administration of iodinated contrast media [1]. Contrast-induced nephropathy (CIN) is most commonly defined as either an absolute (≥ 0.5 mg/dL; ≥ 44 μmol/L) or relative (≥ 25%) increase in serum creatinine levels at 48-72 hours after exposure to iodinated contrast media (CM) [2]. It represents the third cause of acute renal failure in hospitalized patients with an estimated incidence of about 12% [3]. CIN is speculated due to renal damage from acute tubular dysfunction [4] and once the contrast-induced nephropathy is established, there is no definitive treatment, so efforts should be directed toward prevention approaches in order to avoid the event, especially in those with a high risk. There are several studies that analyze the role of multiple prophylactic strategies that have been used to prevent contrast-induced nephropathy and they include volume expansion with sodium chloride or bicarbonate or both, administration of N-acetyl cysteine, statins, hemofiltration or hemodialysis, and reducing the volume of contrast media administered. Despite these varied strategies, there is no clear consensus in clinical practice about the most effective intervention to prevent or reduce this condition. Therefore, further knowledge about the possibility of the presence of this entity by the treating doctor, as a member of a multidisciplinary team including the radiologist, is imperative to distinguish the high-risk factors in patients for developing acute kidney injury (AKI) following parenteral administration of radiocontrast agents.

## Review

The use of contrast media in the diagnostic and therapeutic medical arsenal, like interventional cardiology procedures and computed tomography (CT) scans [5], can cause adverse effects such as renal toxicity and acute kidney injury, known as contrast-induced nephropathy (CIN). The estimated incidence is about 7% to 11%. It is established in the literature that CIN is one of the most important etiological factors of acute kidney injury in hospital settings, and thereby it leads to high health care costs, longer hospital stay, and increased morbidity and mortality [6]. The most recognized definition is an absolute (≥0.5 mg/dl, ≥44 µmol/l) or relative increase (≥25%) in baseline serum creatinine (SCr) value at 48 to 72 hours post-exposure to the contrast media, in the absence of an alternative cause. However, there are many definitions proposed in the world literature. It should be noted that according to the Kidney Disease Improving Global Outcomes guidelines (KDIGO), the recently proposed definition is an increase of ≥ 50% of SCr or ≥ 0.3 mg/dl that usually occurs at 48 hours [7, 8]. In general, the peak of elevation of SCr levels is three to five days post-exposure, returning to normal levels after seven to ten days or even, according to other reports, up to 10-21 days. So contrast-induced nephropathy is considered self-limited and reversible; rarely it causes a persistent renal failure that deserves dialysis [9]. CIN causes kidney damage by acute tubular dysfunction. Many mechanisms are involved in this, namely, renal vasoconstriction [10], toxic effects by the contrast, and oxygen free-radical injury [11]. Once CIN is established, there is no definitive treatment [12], so the most effective strategy remains prevention, especially in those with a high risk of CIN [13].

Promoting factors for the development of nephropathy have been widely described in the literature as well as hydration and pharmacological measures to prevent its development. In this order, with respect to the management, European Renal Best Practice (ERBP) [14] recommends pre-procedure assessment of creatinine concentrations and thus identifying high-risk patients—mostly patients with chronic kidney disease, diabetes mellitus, older age, use of intra-aortic balloon pumps [15], cardiovascular diseases, and people with high C-reactive protein (CRP) levels [16]—to whom a repeat serum creatinine test 12 and 72 hours after administration of the contrast media should be performed. A simple risk score for CIN has been developed for patients undergoing percutaneous coronary intervention (PCI) [17]. This enables the clinician to be prepared for an event if it occurs. On the other hand, it is recommended that clinicians evaluate the benefit: risk ratio of interventions with contrast administration mainly in high-risk patients, consider alternative imaging methods without contrast, with diagnostic accuracy similar to the first. As risk factors of CIN, the volume of contrast applied and the use of concurrent nephrotoxic medication (nonsteroidal anti-inflammatory drugs, aminoglycosides, amphotericin B, high doses of loop diuretics, and antiviral drugs like acyclovir and foscarnet) can be mentioned [18-23]. Therefore, minimizing the volume of CM used and avoiding the use of nephrotoxic medications whenever possible are mandatory prevention measures [24].

Furthermore, an adequate fluid and electrolyte support is one of the most effective prevention strategies for CIN [25]. So, ERBP suggests volume expansion with either isotonic sodium chloride or sodium bicarbonate solutions [26]. Also, the oral route for hydration is possible, as long as an adequate intake of fluid and salt are assured. The mechanism by which it reduces risk is not well understood; however, what is postulated is that the dilution of contrast media may subsequently decrease the nephrotoxicity. Hydration also causes the inhibition of renin-angiotensin-aldosterone system (RAAS) [27], minimizing renal vasoconstriction and hypoxia. When normal saline is used, an intravenous regime of 1.0-1.5 ml/kg/h for at least six hours before and after contrast medium administration is recommended. For sodium bicarbonate [28, 29], the most widely used regimen (3 ml/kg/h for one hour before contrast medium followed by 1 ml/kg/h for six hours after) seems appropriate [30, 31].

In addition to pharmacological prevention strategies, N-acetyl cysteine [32] can be used as a preventive therapy. Its nephroprotective role is attributed to its antioxidant and vasodilatory properties, which increase nitric oxide vasodilator activity [33]); however, this should be used in conjunction with proper volume expansion. Others like theophylline, fenoldopam, and ascorbic acid failed to show any protective effect against CIN [34-37]. Statins have a beneficial role [38, 39] in CIN wherein they improve endothelial dysfunction and decrease oxidative stress, although, there are not enough trials to prove this benefit as conclusive [40-42].

In the same way, hemofiltration has been proven effective in the prevention of CIN [43, 44]; however, future studies are necessary prior to its establishment as a systematic prophylactic measure [45] in patients with kidney injury. Hemodialysis, however, is not recommended [46]. Newer agents like the non-ionic low and iso-osmolar contrast media been developed, which have promised to reduce the incidence of CIN in patients [47].

Contrast-induced nephropathy is a condition in which deterioration of renal function occurs due to exposure to iodinated contrast medium and one that doesn’t have an effective therapy available to treat it. So the best option for the management of CIN is prevention. Despite the existing varied strategies—maintain adequate volume expansion in the peri-procedure period, minimize the volume of contrast media, avoid the use of nephrotoxic medications whenever possible—no clear consensus exists in clinical practice about the most effective intervention to prevent or reduce CIN. Some promising trials are in process that suggest a transradial approach instead of transfemoral access for prevention of CIN, and more evidence on this is yet to be elucidated [48].

## Conclusions

The knowledge of contrast-induced nephropathy is one of the most important points for its prevention, considering that the treating physician is the main link in this chain together with the radiologist and the patient. Consequently, it is expected to minimize the inappropriate use of the contrast media administered in radiological studies.
